# Otitis Media; Its Causes and Treatment

**Published:** 1893-12

**Authors:** Robt. E. Moss

**Affiliations:** San Antonio


For Texas Medical Journal.

### OTITIS MEDIA, ITS CAUSES AND TREATMENT.

ROBT. E. MOSS, M. D., SAN ANTONIO.

THIS SHORT PAPER, with reports of a few cases, is not intended as a scientific one for specialists, but as a practical paper, to impress upon the minds of general practitioners the great importance of this disease, and their duty to their patients. The reason this is written more particularly for the general physician, is the fact that he sees these cases, as a rule, long before the specialists, and in no class of diseases do we find more relief from prompt, early treatment; therefore the importance of seeing these cases before serious pathological changes have occurred.

Otitis media, for practical purposes, may be divided into acute and chronic, suppurative and non-suppurative.

In acute non-suppurative otitis we find, as predisposing causes, former neglected catarrhs of the nose and naso-pharynx, enlargement of third tonsil, and adenoids in vault of pharynx. As exciting causes we have cold, fevers, especially measles, scarlet fever, small pox, etc.; the nasal douche, and teething, are also said by some to be a cause.

The symptoms are deep-seated pain, a sense of fullness, as if the external canal were filled with water, pulsating tinnitus, partial or complete deafness, pain and tenderness on pressure below auricle.

The treatment in this condition is strictly antiphlogistic, and removal of the cause. Leeches in front of tragus, mustard plaster over mastoid, followed by hot fomentations. Just a word as to how heat should be applied: Fill the external canal of the ear with water as hot as can be borne; then apply rubber bag filled with hot water, over the ear, having the patient to lie on well side. In lieu of rubber bag, flax-seed meal or mush poultice, covered with a hot, dry flannel. This should not be continued longer than two or three hours, without allowing an interval of rest, then renew again, if needed. A brisk saline purge is highly useful in these cases; aconite, if there be any fever, and an opiate if pain is excruciating. The nose and naso-pharynx should be carefully cleansed, followed by gentle politzerization. The empiric plan of putting into the external canal irritating substances, as stimulating instillations, only tends to aggravate the disease. Instillations of morphine, atropine, cocaine, etc., should be regarded simply as sedatives, and not as treatment of the disease. These cases are usually treated by physicians as if they were neuralgic instead of inflammatory.

If the membrana tympani is seen to be bulging, and the symptoms do not quickly abate by the above treatment, paracentesis should be performed without delay. It must not be forgotten that in nine out of ten cases, if not the tenth also, the cause of the trouble is closure of the eustachian tubes from enlargement of third tonsil, adenoids, or extension of acute naso-pharyngeal catarrh; therefore, treatment or removal of the cause is imperative to effect a speedy cure.

Acute suppurative otitis, as a rule, follows neglected acute non-suppurative otitis. The causes, therefore, in the main, are the same, and at first the treatment is practically the same. If we suspect pus formation from the throbbing pain, bulging membrane, etc., we should do paracentesis at once in the lower segment, behind the handle of malleus. This operation, though apparently one of the simplest in surgery, requires a practiced hand and eye. Careful treatment of nose and naso-pharynx, Politzer inflation, cleansing and drying of external canal, followed by insufflations of boric acid, or, what I prefer, a solution of same in alcohol and glycerine, put in quite warm. This, in a few words, is the treatment for the majority of acute cases; and if, in connection with good hygiene and constitutional treatment, it is intelligently and faithfully carried out, the greater number will end in complete recovery, with little, if any, impairment of hearing.

Chronic suppurative otitis is simply a continuation of the acute suppurative cases, either from neglect or improper treatment, or extension to the mastoid, and caries of the ossicles.

These cases come to us with the statement that they have a "running from the ears;" and aside from that there are few, if any, symptoms which belong to the chronic type.

We usually find caries of the bones, mastoid disease, polypoid tissue, or cholesteatoma, the cause of the discharge of pus. Closure of the eustachian tubes by pressure of adenoids, or third tonsil, will keep up a muco-purulent discharge indefinitely. Treatment in these cases is almost entirely surgical; removal of the cause being sufficient, in the greater proportion of cases, to effect a cure. This should be followed by cleansing out the discharge with peroxide of hydrogen, drying the canal, and insufflating iodoform, if due to caries; if not, boric acid, or some mild astringent solution. Here, as elsewhere, the cause must be found and removed, then the discharge will cease, if the parts are simply kept clean.

We come now to non-suppurative otitis media. Nasal obstructions due to posterior hypertrophies of inferior turbinated bones, spur on septum, deviations of septum, chronic catarrhs, etc., are the most frequent causes of this form of otitis. It occasionally follows acute cases, especially where they are neglected. There are no pronounced symptoms in this form of otitis; the onset is so insidious that patients rarely apply for treatment until hearing is markedly impaired, or there is distressing tinnitus. The majority of cases of deafness occurring in middle life are due to this condition, and not "inherited," as some physicians and the laymen believe. We do not see these cases, as a rule, until the golden time for treatment has passed, unless we systematically examine the ears of every patient applying to us for any form of nose or throat trouble. Treatment, however, is highly beneficial for a great number, but it is necessarily prolonged; and few patients will continue treatment longer than three to six months, when one to three years is often necessary to accomplish any permanent relief. The general practitioner does absolutely nothing for these cases, and they are the *bete noir* to the majority of specialists. The nose and naso-pharynx should receive careful attention; then inflating the middle ear with fumes of iodine, menthol, camphor, carbolic acid, etc., preferably with the catheter and a douch apparatus. Great care should be exercised in cleansing the nose, then wiping out with absorbent cotton, before introducing the catheter, or some mucus from the nose might be forced into the middle ear, causing serious mischief. The treatment must be varied, and remedies selected to meet the conditions found. If the drum membrane is found retracted, opaque, and free from inflammation, yet showing a thickened condition, and ossicles partially anchylosed, I find inflations of iodine fumes, with gentle massage by means of Siegle's pneumatic speculum, gives the most satisfactory results. If the tubes are occluded, apply nitrate of silver, forty grains to the ounce, or ten per cent. of iodine in glycerine, around the mouth of tubes, in connection with the forcible use of the Politzer bag; this will usually succeed. I am opposed to the use of the filiform bougie to overcome constrictions; it is liable to wound the delicate membrane, and the cicatricial contraction cause permanent stricture. The condition of the stomach and bowels should receive careful attention in this class of cases, for there is no question that irritating eructations will increase an existing pharyngitis, and secondarily the lining membrane of tubes and middle ear.

Where there is marked retraction of the drum membrane, anchylosis of ossicles, with persistent and distressing tinnitus, the only plan of relief is by removal of the ossicles. This operation is almost devoid of danger, when carefully done, and the results highly satisfactory in certain cases.

The following cases are taken from my case-book, and will illustrate the benefits of early treatment:

Katie Y., age 7. Seen first March 20, 1893. Her mother brought her, as she said, on account of "sniffling" and mouth breathing. Her facial expression was typical of adenoids, and examination revealed the vault of pharynx filled with them, tonsils enlarged and follicular, external ear full of impacted cerumen. Tuning fork heard much louder over mastoids, watch by pressure. Cerumen removed, but hearing not improved. Advised early operation for removal of tonsils and adenoids. One week later performed operation under chloroform. After treatment, cleansing spray, with good iron tonic. Three weeks later she could hear watch with either ear forty inches, mouth breathing had ceased, and she was much improved every way.

Charlotte T., age 7. Saw her first July 21, 1893. She complains that her nose is always "stopping up," breathes through her mouth at night, takes cold easily, and appetite very capricious. Examination showed enlarged tonsils, and vault literally filled with adenoids. Hearing watch—right, one inch; left, eight inches. Operated on the 24th; after treatment the same as above. Three weeks later, hearing normal in both ears; no mouth breathing at night.

November 28, 1892. Edith M. and Ruby M., sisters, 7 and 4 years old. History of repeated attacks of acute ear-ache, followed by discharge from both ears of each little patient. Examination showed perforations in each ear of both, adenoids and enlarged tonsils. They looked pale, poorly nourished, listless, and almost stupid. Operated on both for adenoids and enlarged tonsils, gave cleansing spray for nose, had their ears carefully syringed twice daily, and put in ten drops of a solution of boric acid in alcohol and glycerine. Gave a tonic of iron and cod-liver oil, beef and milk diet, with instructions to live in the open air. One month later perforations healed, discharge had ceased, hearing practically normal. The older one had gained five, and the younger, four pounds; cheeks rosy, and seemingly in perfect health.

December 10, 1893. Edward T., age 22 months. Father brought him for ear-ache and constantly recurring discharge of pus from both ears. Examination after careful syringing revealed perforation in Shrapnell's membrane of each ear. Examining vault of pharynx with finger, found mass of adenoid tissue, which was removed at once with curette. One week after, discharge has ceased and perforations closed. The little fellow (as do the others reported) continues well at this time.

I will refrain from reporting any chronic cases, as it would extend this paper beyond reasonable length.

It is not surprising that these cases are neglected, when such an eminent specialist as Dr. H. Knapp had never examined or operated on a case of adenoids prior to 1892. In these conditions there is nearly always an inherited syphilitic or tubercular diathesis from parents or grandparents; therefore constitutional treatment is demanded in nearly all cases. The importance of an intimate knowledge of general diseases cannot be overestimated, for as Macdonald has truly said: "Specialism is a scientific absurdity, although a practical necessity."

If this should be the means of causing even a few physicians not to neglect the little ones with ear-ache or suppuration (by telling the anxious mother "they will outgrow it"), but to refer them to some specialist, if not prepared to treat them properly, I shall feel that it has not been written in vain.

